# Adventures of a Female Medical Detective: In Pursuit of Smallpox and AIDS

**DOI:** 10.3201/eid2208.160532

**Published:** 2016-08

**Authors:** Polyxeni Potter

**Affiliations:** Founding Managing Editor, Emerging Infectious Diseases Journal, Atlanta, Georgia, USA

Light-hearted and easy to read, Mary Guinan’s account of her career as an epidemiologist accomplishes its goal, “To help readers better understand and value the public health system that exists for the protection of the nation’s health and for the prevention of disease and injury.” From the early definition of the “hole in the sole,” the epidemiologist’s trademark, to the description of the Sherlock Holmes method, the epidemiologist’s approach to public health puzzles, Guinan’s stories embody the modesty and humor inherent in the culture of epidemiology as practiced by the Epidemiologic Intelligence Service (EIS) of the Centers for Disease Control and Prevention.

Young readers will be surprised, indeed as Guinan herself was surprised, at the challenges posed each day in public health, and in the end, they too may “find something to believe in,” as she did during her days in the Smallpox Eradication Program. Her colleagues, EIS alumni and those now in the EIS Program, will see themselves in her description of the demands for instant, on the spot, expertise on complicated health emergencies or unknown sexually transmitted infections. They will recognize the unlikely developments so much a part of human interaction during epidemiologic investigations: pink elephants, unplanned media presentations, expert witnesses, hostile refugee camps, veiled women, seedy hotels, life-threatening needlestick injuries, famous movies featuring you, a challenging milk industry.

Investigation surprises abound in Dr. Guinan’s account. Some involve the investigator herself. “I don’t think anyone grows up wanting to be a physician who specializes in sexually transmitted diseases.” “I was never the most important leader. A medical detective has a small part in a team effort, usually a very large team… the medical detectives who collect clues, analyze data, investigate suspected cases, and carry out their public health mission.”

Some surprises involve the context of the investigation: for example, the Mujahideen in an Afghan refugee camp asking Dr. Guinan for weapons to fight in the Haj in 1980. She worked in the cramped quarters of a hotel in the Tenderloin District of San Francisco, identifying what would eventually be called the first AIDS cases. She later had to explain to the public that “nice” women can get AIDS.

Guinan and Mather wrote a book that is readable and free of technical jargon. The book is a woman’s account because it reflects the experiences of a woman traveling the paths women have not traditionally traveled. During Guinan’s first outbreak investigation, “I walked up to the uniformed four men and one woman and identified myself. The men were clearly shocked that ‘Dr. Guinan’ was a woman, and they were apologetic. They had not known that CDC was sending a woman.”

